# Integrating Tobacco Treatment Into Oncology Care: Reach and Effectiveness of Evidence-Based Tobacco Treatment Across National Cancer Institute–Designated Cancer Centers

**DOI:** 10.1200/JCO.22.00936

**Published:** 2022-12-06

**Authors:** Sarah D. Hohl, Richard S. Matulewicz, Ramzi G. Salloum, Jamie S. Ostroff, Timothy B. Baker, Robert Schnoll, Graham Warren, Steven L. Bernstein, Mara Minion, Katie Lenhoff, Neely Dahl, Hee Soon Juon, Ursula Tsosie, Linda Fleisher, Heather D'Angelo, Alex T. Ramsey, Kimlin T. Ashing, Betsy Rolland, Margaret B. Nolan, Jennifer E. Bird, Claire V.T. Nguyen, Danielle Pauk, Robert T. Adsit, Hilary A. Tindle, Kimberly Shoenbill, Sophia Yeung, Cary A. Presant, Kara P. Wiseman, Kuang-Yi Wen, Lou-Anne Chichester, Li-Shiun Chen

**Affiliations:** ^1^ Carbone Cancer Center, School of Medicine and Public Health, University of Wisconsin-Madison, Madison, WI; ^2^ Department of Family Medicine, School of Medicine and Public Health, University of Wisconsin-Madison, Madison, WI; ^3^ Memorial Sloan Kettering Cancer Center, Department of Surgery, Urology Service, New York, NY; ^4^ Department of Health Outcomes and Biomedical Informatics, University of Florida College of Medicine, and University of Florida Health Cancer Center, Gainesville, FL; ^5^ Department of Psychiatry & Behavioral Sciences, Memorial Sloan Kettering Cancer Center, New York, NY; ^6^ Center for Tobacco Research and Intervention, Department of Medicine, University of Wisconsin School of Medicine and Public Health, Madison, WI; ^7^ Department of Psychiatry and Abramson Cancer Center, University of Pennsylvania, Philadelphia, PA; ^8^ Department of Radiation Oncology, College of Medicine, Medical University of South Carolina, Charleston, SC; ^9^ Department of Emergency Medicine, C. Everett Koop Institute, Norris Cotton Cancer Center, Geisel School of Medicine, Dartmouth College, Hanover, NH; ^10^ Dartmouth-Hitchcock Norris Cotton Cancer Center, Geisel School of Medicine, Dartmouth College, Hanover, NH; ^11^ University of Virginia Cancer Center, Charlottesville, VA; ^12^ Department of Medical Oncology, Thomas Jefferson University, Philadelphia, PA; ^13^ Seattle Cancer Care Alliance, Seattle, WA; ^14^ Fox Chase Cancer Center, Philadelphia, PA; ^15^ Alvin J. Siteman Cancer Center at Barnes-Jewish Hospital and Washington University School of Medicine, St Louis, MO; ^16^ Department of Psychiatry, Washington University School of Medicine, St Louis, MO; ^17^ Department of Population Sciences, City of Hope National Medical Center, Duarte, CA; ^18^ Institute for Clinical and Translational Research, School of Medicine and Public Health, University of Wisconsin-Madison, Madison, WI; ^19^ Center for Tobacco Research and Intervention, School of Medicine and Public Health, University of Wisconsin-Madison, Madison, WI; ^20^ Department of Medicine, Vanderbilt University School of Medicine, Nashville, TN; ^21^ Geriatric Research Education and Clinical Centers (GRECC), Veterans Affairs Tennessee Valley Healthcare System, Nashville, TN; ^22^ Department of Family Medicine, Lineberger Comprehensive Cancer Center, University of North Carolina-Chapel Hill, Chapel Hill, NC; ^23^ Department of Supportive Care Medicine, City of Hope National Medical Center, Duarte, CA; ^24^ Department of Medical Oncology and Therapeutics Research, City of Hope National Medical Center, Duarte, CA; ^25^ Department of Public Health Science, University of Virginia School of Medicine, Charlottesville, VA

## Abstract

Quitting smoking improves patients' clinical outcomes, yet smoking is not commonly addressed as part of cancer care. The Cancer Center Cessation Initiative (C3I) supports National Cancer Institute–designated cancer centers to integrate tobacco treatment programs (TTPs) into routine cancer care. C3I centers vary in size, implementation strategies used, and treatment approaches. We examined associations of these contextual factors with treatment reach and smoking cessation effectiveness.

**METHODS:**

This cross-sectional study used survey data from 28 C3I centers that reported tobacco treatment data during the first 6 months of 2021. Primary outcomes of interest were treatment reach (reach)—the proportion of patients identified as currently smoking who received at least one evidence-based tobacco treatment component (eg, counseling and pharmacotherapy)—and smoking cessation effectiveness (effectiveness)—the proportion of patients reporting 7-day point prevalence abstinence at 6-month follow-up. Center-level differences in reach and effectiveness were examined by center characteristics, implementation strategies, and tobacco treatment components.

**RESULTS:**

Of the total 692,662 unique patients seen, 44,437 reported current smoking. Across centers, a median of 96% of patients were screened for tobacco use, median smoking prevalence was 7.4%, median reach was 15.4%, and median effectiveness was 18.4%. Center-level characteristics associated with higher reach included higher smoking prevalence, use of center-wide TTP, and lower patient-to-tobacco treatment specialist ratio. Higher effectiveness was observed at centers that served a larger overall population and population of patients who smoke, reported a higher smoking prevalence, and/or offered electronic health record referrals via a closed-loop system.

**CONCLUSION:**

Whole-center TTP implementation among inpatients and outpatients, and increasing staff-to-patient ratios may improve TTP reach. Designating personnel with tobacco treatment expertise and resources to increase tobacco treatment dose or intensity may improve smoking cessation effectiveness.

## BACKGROUND

Nearly 25% of patients with cancer report current smoking at the time of diagnosis.^1^ Quitting smoking after diagnosis improves patients' first-line treatment response, health-related quality of life, and overall survival,^2,3^ and reduces likelihood of a second primary tumor.^4^ Despite the National Comprehensive Cancer Network clinical practice guidelines for smoking cessation^5^ and the National Cancer Institute (NCI), Commission on Cancer, and American Association for Cancer Research recommendations to include screening for and treating tobacco use as a quality measure,^6^ smoking is not commonly addressed as central to cancer care.^7^ The Cancer Center Cessation Initiative (C3I), launched in 2017 as part of the Cancer Moonshot program, supports NCI-designated cancer centers to integrate evidence-based tobacco treatment into routine cancer care.^8^ C3I focuses on implementing evidence-based tobacco treatment, and its evaluation is guided by the reach, effectiveness, adoption, implementation, and maintenance (RE-AIM) framework.^9,10^ Key implementation outcomes including tobacco treatment reach and smoking cessation effectiveness are reported by centers twice annually. Since 2017, C3I centers have successfully integrated tobacco treatment into routine oncology care.^11^ These efforts have increased *tobacco treatment reach*, defined as patients' receipt of one or more components of evidence-based tobacco treatment, such as counseling, pharmacotherapy, and referrals to quitlines.^12^

**CONTEXT**

**Key Objective**
Identify implementation strategies and evidence-based tobacco use treatment components associated with high rates of treatment reach and smoking cessation effectiveness across National Cancer Institute–designated cancer centers participating in the Cancer Center Cessation Initiative.
**Knowledge Generated**
Higher center-level smoking prevalence, use of center-wide tobacco treatment program (compared with partial center programming), and lower patient-to-tobacco treatment specialist ratio were associated with better treatment reach. Better smoking cessation effectiveness was observed at centers that served a larger overall patient population, served a larger population of patients who smoke, reported a higher smoking prevalence, and/or offered electronic health record referrals via a closed-loop system.
**Relevance *(S.B. Wheeler)***
This study provides an excellent summary of contextual characteristics and implementation strategies associated with greater reach and effectiveness among National Cancer Institute–designated cancer centers participating in tobacco cessation initiatives and should inform programming at centers going forward.*
*Relevance section written by *JCO* Associate Editor Stephanie B. Wheeler, PhD, MPH.

C3I seeks to advance understanding of ways to improve access and utilization of tobacco treatments in real-world cancer care settings.^10^ Its goal is to enhance the population impact of evidence-based tobacco treatment program (TTP) interventions, particularly in settings with low treatment reach.^13^ C3I provides the unique opportunity to identify characteristics and implementation strategies that demonstrate success with regard to treatment reach and smoking cessation effectiveness. C3I centers apply multiple implementation strategies in settings varying in size, smoking prevalence, and resource availability, thus offering an unprecedented opportunity to examine factors associated with tobacco treatment reach and smoking cessation effectiveness among patients with cancer. Here, we (1) assess rates of treatment reach and smoking cessation effectiveness by cancer center and TTP characteristics and (2) identify implementation strategies and evidence-based tobacco treatment components associated with high treatment reach and smoking cessation effectiveness.

## METHODS

The C3I Coordinating Center at the University of Wisconsin Carbone Cancer Center administers a web-based Qualtrics survey to C3I centers every 6 months. Survey questions assess center characteristics and implementation outcomes including treatment reach, and smoking cessation effectiveness. Each center selected implementation workflow and strategies that best suited their resources and patient populations. Methods for measuring treatment reach and smoking cessation effectiveness at the center level have been described elsewhere.^11,14^ This cross-sectional study uses 6-month data from 28 NCI-designated cancer centers in C3I that submitted a report for the January-June 2021 reporting period. This reporting period was selected to maximize data quality; it was the most recent reporting period and included treatment reach and smoking cessation effectiveness data from the greatest number of C3I centers since the initiative's inception. To be included in the analysis, centers must have reported both treatment reach and smoking cessation effectiveness data and have received C3I funding as part of cohort 1 (n = 22 total centers funded from 2017 to 2019) or cohort 2 (n = 20 centers funded from 2018 to 2020). Cohort 3 centers were funded in late 2020 and most did not have 6-month follow-up data available for the January-June 2021 reporting period. The study was categorized as program evaluation and deemed exempt by the University of Wisconsin-Madison Institutional Review Board.

### Implementation Outcomes: Treatment Reach and Smoking Cessation Effectiveness

*Treatment reach* (referred to as *reach* throughout) is defined as the proportion of unique patients seen during the 6-month reporting period who reported current smoking and who received at least one evidence-based tobacco treatment component (eg, point-of-care counseling, telephone-based counseling, pharmacotherapy, or quitline referral). The denominator for treatment reach was the number of patients seen at each C3I center during the 6-month reporting period who were identified as currently smoking. *Smoking cessation effectiveness* (referred to as *effectiveness* throughout) was defined as the proportion of patients currently smoking who reported 7-day point prevalence abstinence 6 months following receipt of evidence-based tobacco treatment. Centers applied different approaches to conduct follow-up with patients, including automated (ie, interactive voice response) or staff-initiated phone calls, data extracted from the electronic health record (EHR), patient portals, and manual chart reviews. We used an intention-to-treat approach to assess effectiveness, in which all patients who received or were referred to treatment were included in the effectiveness analysis. Two centers were unable to estimate how many patients were missing follow-up data; those centers' data were analyzed using a complete response approach.

### C3I Center Characteristics, Tobacco Treatment Program Characteristics, and Implementation Strategies

We assessed the following cancer center characteristics: size (total number of unique adult patients), number of adult patients who reported current smoking, and center-level smoking prevalence. We assessed three TTP program characteristics: (1) clinical setting (cancer center-wide; part of cancer center [eg, inpatient or outpatient only]); (2) targeted patients selected for TTP engagement (inpatient and outpatient); and (3) number of patients who reported smoking per 1.0 full-time equivalent (FTE) tobacco treatment specialist (TTS). Then, we assessed two implementation strategies: (1) referral mechanism (EHR-based with or without closed-loop) and (2) billing practices (billed for medication, counseling, both, or none). EHR referrals (eReferrals) included patient referrals to tobacco treatment that were generated electronically. In a closed-loop EHR referral system, the EHR is populated with referral outcomes (eg, receipt of treatment and patient abstinence) from an outside tobacco cessation service vendor (eg, a quitline).

### Evidence-Based Treatment Components

We assessed delivery of eight evidence-based tobacco treatment components (listed in Table 1). Point-of-care treatment included brief advice to quit smoking and discussion of evidence-based tobacco treatment options^15^ delivered by a frontline oncology care provider (eg, oncologist or nurse) during routine oncology appointments. In-person counseling provided by a provider other than a frontline oncology provider (eg, TTS or psychologist) was captured in the in-person face-to-face counseling response. The Data Supplement (online only) lists the survey questions analyzed for this manuscript.

**TABLE 1.**
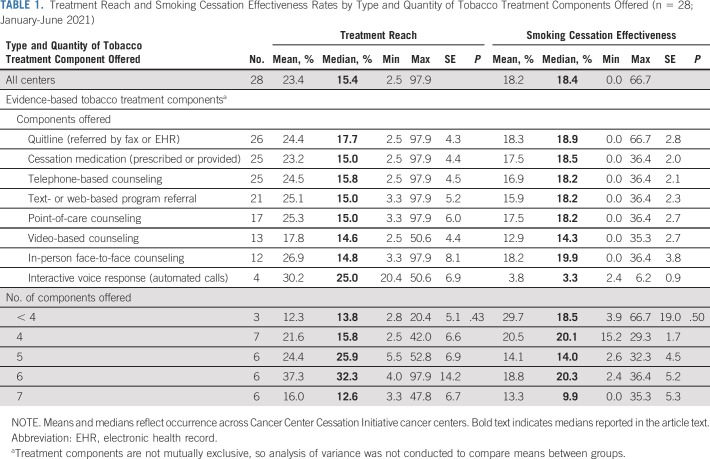
Treatment Reach and Smoking Cessation Effectiveness Rates by Type and Quantity of Tobacco Treatment Components Offered (n = 28; January-June 2021)

### Analysis

Data were analyzed using SPSS (Version 27). To assess center-level reach and effectiveness, we computed medians and means and summarized descriptive comparisons overall and by center characteristics, implementation strategies, and type and number of evidence-based treatment components offered. This approach assumed equal weight to each center regardless of size. Differences in reach and effectiveness means were examined using analysis of variance. We report both mean and medians in tables; however, we highlight the medians in the text and figures since it is a more appropriate measure of central tendency, given the wide variation in reach and effectiveness across C3I centers. To identify potential bias in assessing the relationship between each covariate and rates of reach and effectiveness, we examined associations among center-level characteristics and implementation strategies. We noted positive associations as potential confounders in the relationship between a given covariate and rates of reach and effectiveness. We then plotted mean reach and effectiveness across cancer center characteristics and TTP implementation strategies.

## RESULTS

### C3I Center Characteristics

**FIG 1.**
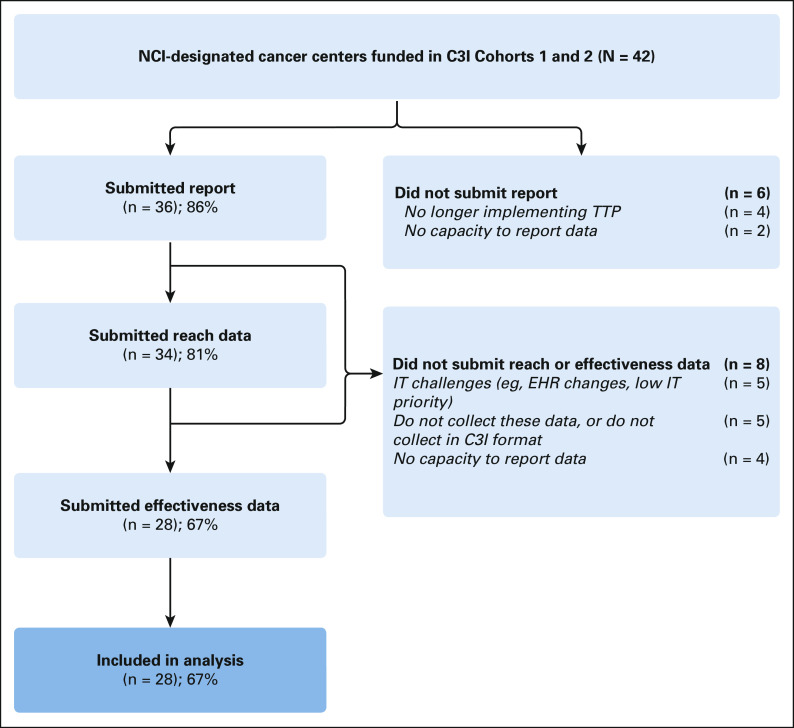
Flowchart depicting the inclusion criteria in the analysis. C3I, Cancer Center Cessation Initiative; EHR, electronic health record; IT, information technology; NCI, National Cancer Institute; TTP, Tobacco Treatment Program.

**FIG 2.**
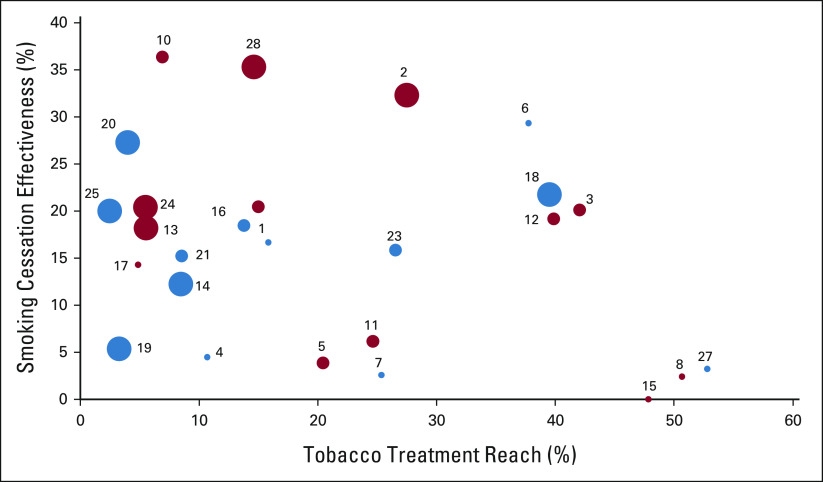
Rates of tobacco treatment reach and smoking cessation effectiveness among patients at NCI-Designated Cancer Centers in the Cancer Center Cessation Initiative by cancer center characteristics (n = 28; January-June 2021). NCI, National Cancer Institute.

**FIG 3.**
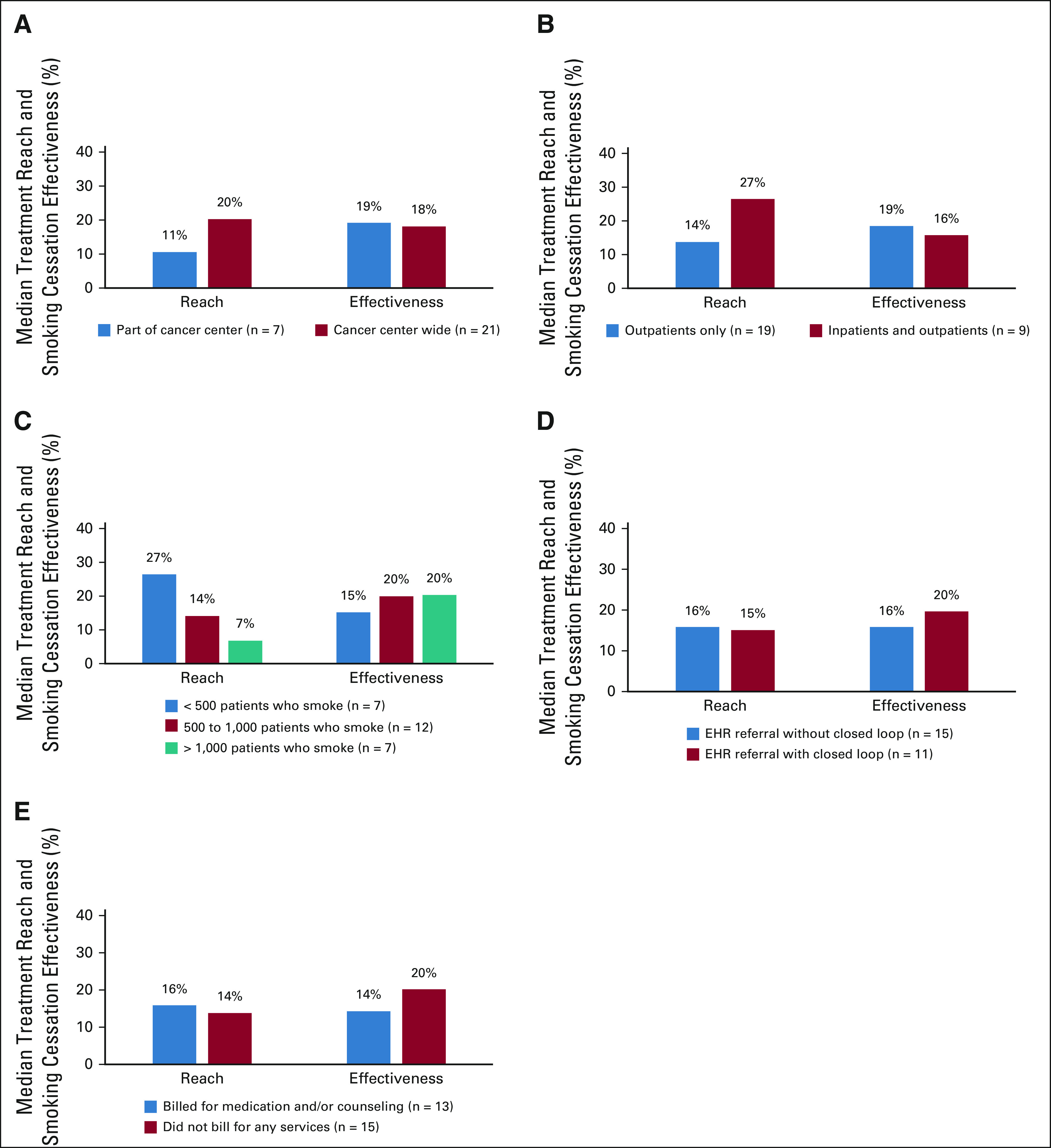
Median treatment reach and smoking cessation effectiveness by tobacco treatment by program characteristics and implementation strategies (n = 28 centers; January-June 2021): (A) cancer center setting, (B) patients eligible, (C) number of patients per TTS, (D) referral mechanism, and (E) billing practices.

A total of 28 (67%) cohort 1 and 2 centers were included in the analysis (Fig 1). Between January and June 2021, 692,662 unique adult patients received cancer care across centers (center-level median: 20,509; Table 2). Center-level median screening rate for smoking was 95.5%; median smoking prevalence was 7.4%. Of patients screened across all centers, 44,437 reported current smoking (median: 1,004). Centers employed a median of 1.0 TTS FTE for every 682 patients who smoked. Center-level median reach was 15.4% and median effectiveness was 18.4%, but centers demonstrated variation in reach and effectiveness by center size and smoking prevalence (Fig 2). Smaller centers (< 12,000 patients) achieved reach rates above the median but effectiveness rates below the median. Larger cancer centers (> 27,500 patients) achieved reach rates below the median but effectiveness rates above the median (Table 3). Similarly, centers with larger populations of patients who smoked had lower reach but higher effectiveness. Centers with higher smoking prevalence had higher reach (22.5% *v* 14.2%) and effectiveness (19.7% *v* 16.3%) than those with lower smoking prevalence (Fig 3).

**TABLE 2.**
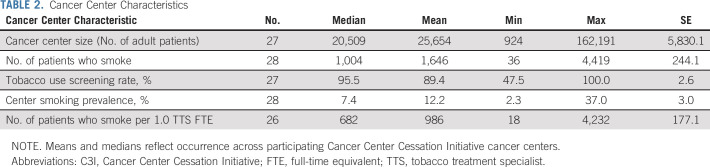
Cancer Center Characteristics

**TABLE 3.**
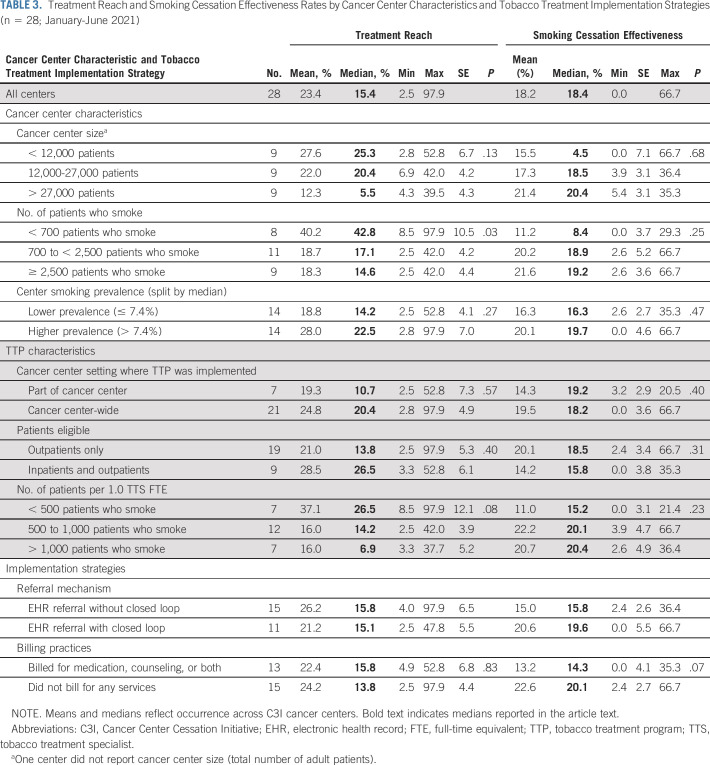
Treatment Reach and Smoking Cessation Effectiveness Rates by Cancer Center Characteristics and Tobacco Treatment Implementation Strategies (n = 28; January-June 2021)

### TTP Program Characteristics and Implementation Strategies

Compared with the 7 (25%) centers that implemented the TTP in part of the center, 21 implemented the TTP center-wide, a strategy associated with higher median reach (20.4% *v* 10.7%) and similar effectiveness (18.2% *v* 19.2%; Table 3). Centers at which only outpatients were targeted for the TTP (n = 19) had lower median reach compared with those at which both outpatients and inpatients were targeted (13.8% *v* 26.5%) but slightly higher effectiveness (18.5% *v* 15.8%). All but two centers (93%) employed one or more TTSs. Centers with < 500 patients who smoked per 1.0 TTS FTE had higher median reach (26.5%) but lower effectiveness (15.2%) than those with ≥ 500 patients who smoked per 1.0 TTS FTE. Most (93%) centers used an eReferral system; median reach was similar for those centers regardless of whether the referral was implemented with or without closed-loop (15.1% *v* 15.8%) but effectiveness was higher for centers that used a closed-loop system (15.8% *v* 19.6%). The 13 (46%) centers that billed for medication, counseling, or both had higher median reach (15.8%) compared with those that did not bill for any services (13.8%), but lower effectiveness (14.3% *v* 20.1%).

### Evidence-Based Tobacco Treatment Components

All but three centers offered four or more tobacco treatment components, but rates of treatment reach and smoking cessation effectiveness did not differ significantly on the basis of number of components offered. The most commonly offered components included quitline referrals (93%), pharmacotherapy (89%), text or web-based program referrals (75%), and counseling by telephone (89%) or point-of-care (61%; Table 1). Only four (14%) centers offered treatment via interactive voice response, which had the highest median reach (25.0%) but the lowest effectiveness (3.3%) of any component offered. Centers that offered quitline referrals had the second highest median reach (17.7%) and effectiveness (18.9%), whereas centers that offered pharmacotherapy, telephone-based counseling, text- and web-based program referrals, and/or point-of-care counseling had similar rates of reach and effectiveness. Centers that offered in-person counseling had comparably lower median reach (14.8%) but the highest effectiveness of all components (19.9%). Centers that offered a variety of six different evidence-based tobacco treatment components had the highest median reach (32.3%) and effectiveness (20.3%).

## DISCUSSION

In this cross-sectional study, we assessed treatment reach and smoking cessation effectiveness for patients with cancer implemented across 28 NCI-designated cancer centers. We found an inverse relationship between reach and effectiveness for several cancer center and TTP characteristics but not for implementation strategies. For example, smaller centers (< 12,000 patients) achieved reach rates above the median but effectiveness rates below the median, and larger centers (> 27,500 patients) achieved reach rates below the median but effectiveness rates above the median. Centers with larger populations of patients who smoked had lower reach but higher effectiveness than those with smaller populations of patients who smoked. This observed inverse relationship between reach and effectiveness by center characteristics may be due to centers with lower reach having fewer patients with whom to follow-up or could be a result of dose or intensity of treatment components received. Alternatively, centers with lower reach may be providing services to patients who are more receptive to treatment and more likely to achieve abstinence. Including more patients who were difficult to refer and treat—resulting in higher treatment reach rates—might have resulted in reaching patients less likely to achieve abstinence, resulting in the lower smoking cessation effectiveness rates observed. Future research should more explicitly assess characteristics of patients referred and evaluate approaches that may better reach and engage patients reticent to participate in tobacco treatment.

These findings can help oncology settings determine if their quality improvement priority is to increase reach or effectiveness and implement strategies that align with those outcomes. Moreover, these findings are important for considering resource allocation. For example, centers with relatively low reach but relatively high effectiveness may prioritize strategies that were found to enhance reach (eg, targeting inpatients and outpatients and increasing staff-to-patient ratios). Centers with relatively high reach but relatively low effectiveness may consider adding designated personnel with tobacco treatment expertise or resources to increase tobacco treatment dose or intensity.

To achieve meaningful clinical impact, TTPs must be designed to reach a high proportion of patients who smoke. The median reach of 15.4% in our study is similar to the 15% average reach reported in primary care settings.^16^ The 18.4% median effectiveness rate in C3I is well above the 15.2% average effectiveness found across 67 clinical trials in diverse care settings.^17^ The lowest rates of reach observed at the largest cancer centers suggest that greater attention is needed to scale up services, identify multilevel barriers, and address tobacco treatment care delivery needs of high-volume centers.

TTPs offered cancer center-wide had higher reach and comparable effectiveness to TTPs offered in part of the center, suggesting that center-wide adoption may result in better implementation outcomes. Centers in which inpatients and outpatients were eligible for the TTP had higher reach but lower effectiveness than those in which only outpatients were eligible, possibly because of inpatients' advanced illness, lower quality of life,^18^ and receipt of end-of-life care.^19^ More research is needed to assess the extent to which expanding TTPs to inpatient settings and coordination of postdischarge tobacco treatment affects reach and effectiveness.

Our results suggest that a higher TTP-to-patient ratio improves reach, findings supported by other studies that have found that employing TTSs who provide high-intensity evidence-based tobacco treatment through multiple modalities (eg, telephone or in-person counseling, and medication management) increases TTP reach.^20^ Centers that had a higher TTS-to-patient ration had lower effectiveness. This finding is surprising given evidence that counseling delivered by cessation specialists improves quit rates.^21^ However, when paired with pharmacotherapy, counseling delivered by any clinician (eg, TTSs, nurses, or psychologists) enhances quit rates,^22^ suggesting that centers may consider developing a collaborative care model to leverage the support of oncology staff at point-of-care without relying solely on TTS support.^15,23^ Median reach was slightly lower among the 54% of centers that did not bill for any tobacco treatment compared with the 46% that did bill. Future research should investigate whether adequate reimbursement for TTPs, including the ability of counselors to bill independently, is associated with implementation outcomes.

All but two C3I centers implemented TTP referrals via the EHR, compared with half or fewer of centers who used this approach 2 years before.^14^ Moreover, the 40% of centers that used a closed-loop system had similar reach and higher effectiveness than those without closed-loop, an implementation strategy associated with increased referrals, documentation of outcomes, and smoking cessation.^24^ Although our assessment approach made it difficult to determine which centers used an opt-out EHR referral approach, the opt-out strategy—in which patients eligible for the TTP are automatically identified and referred to tobacco treatment regardless of willingness to quit—is feasible in cancer care settings^25^; is associated with improved reach, effectiveness, and other related health outcomes^26,27^; and is a promising implementation strategy to improve treatment reach and smoking cessation effectiveness in oncology settings.

Reach can be enhanced with delivery of multiple tobacco treatment modalities, such as pharmacotherapy combined with expanded counseling options, including quitline referrals, telephone, face-to-face, telehealth, and point-of-care counseling. Point-of-care counseling, in which patients are provided a brief intervention or advice by frontline oncology care providers during routine oncology appointments, represents an option that could be used alongside or instead of a referral-out approach. This EHR-enabled approach has demonstrated success in improving rates of screening, referral, and receipt of pharmacotherapy for patients who smoke,^23^ as well as treatment effectiveness and program sustainability during the COVID-19 pandemic.^15^

As an implementation initiative, C3I benefits from data collected across diverse real-world oncology care and geographic settings. The 28 centers included here represent 40% of all NCI-designated cancer centers in the United States with clinical programs; no data set that we know of includes this type of information from as many cancer centers. Yet, several limitations should be noted. First, the findings from NCI-designated cancer centers may not generalize to all community oncology settings where more than 80% of patients receive cancer treatment.^28^ Although the current findings may inform adoption of best practices among community oncology settings, research in those programs is warranted. Just ASK, a 2022 Commission on Cancer quality improvement initiative, which incentivizes cancer centers that assess smoking status among patients newly diagnosed with cancer,^29^ offers an opportunity to build on lessons learned in C3I to address implementation challenges in more heterogenous community cancer care settings. Second, the potential bias in our smoking cessation effectiveness measure must be noted. We used an intention-to-treat approach for the 26 centers that provided their denominator for effectiveness, but a complete response approach for two centers that could not estimate the number of patients missing from 6-month follow-up data. Each center determined its own denominator on the basis of its practices. Systematic assessment of smoking cessation effectiveness in clinical populations using the EHR is still in its infancy and not part of routine care. For example, at some centers, the effectiveness measure was severely limited because the centers did not have access to follow-up data on many patients reached by the TTP. In some cases, institutional policies restrict who can be contacted; in others, patients must opt-in to receive follow-up phone calls. Although recommendations for assessing tobacco use in cancer clinical trials exist,^30^ harmonization and standardization of measurement and documentation of smoking status in cancer registries such as the NCI-supported North American Association for Central Cancer Registries (NAACCR) would greatly facilitate identification of current smokers, promote access to best practices for tobacco treatment, and greatly improve data quality for cancer surveillance and epidemiologic research focusing on the risks of persistent smoking for patients with cancer.

The relationships reported in this paper could be compromised by confounding because of measured or unmeasured variables. For example, programs differ across several measured variables that are likely intercorrelated (eg, center size, number of patients who smoke, types and number of intervention components, and staff ratios). Future sensitivity analyses must be conducted to determine the extent to which associations with outcomes are related to a particular variable independent of other variables with which it is correlated.^31^ This would clarify the nature of the relationships reported here.

Our sample size of 28 centers was too small to detect statistical significance, and it is difficult to determine robust and meaningful differences. Moreover, effectiveness data are difficult to obtain, particularly among patients with cancer who may either be too ill to respond to 6-month follow-up data or may have died. In keeping with a core mission of C3I to integrate and expand TTPs, centers reported allocating more resources to treat tobacco dependence initially rather than follow-up with patients, which could explain the inverse relationship between treatment reach and smoking cessation effectiveness. The aggregate-level data obtained in C3I do not allow for detailed examination of patient-level factors as they relate to outcomes. Demonstrating success and return on investment are critical to sustaining implementation of evidence-based practices.^32,33^ Future hybrid effectiveness-implementation trials should investigate stepped care strategies for scaling up tobacco treatment services ranging from low-burden, low-cost strategies to more intensive multicomponent tobacco treatment counseling and pharmacotherapy to support patients' quit attempts and to maintain smoking abstinence. Longitudinal research examining change in reach and effectiveness over time, and the association of these changes with implementation strategies could identify temporal effects of implementation strategies on reach and effectiveness. Finally, as an observational study, there is no random assignment and the sampling strategy for outcome data is likely biased.

In conclusion, NCI-designated cancer centers have become highly engaged in assessing patient tobacco use. Understanding treatment reach and smoking cessation effectiveness will help guide cancer centers and community oncology practices to select and implement evidence-based interventions and strategies that fit the needs and resources available and improve patient outcomes. However, continued innovative efforts are needed to identify optimal strategies to engage patients in tobacco treatment, to determine which patients would benefit from more intensive tobacco treatment interventions, and to improve treatment reach and smoking cessation effectiveness and its reporting.
